# Compliance assessment regarding the PVC management on normal wards of a university hospital

**DOI:** 10.3205/dgkh000430

**Published:** 2023-01-27

**Authors:** Volha Rusinovich, Yury Rusinovich, Iris F. Chaberny, Susanne Kolbe-Busch

**Affiliations:** 1 Institute of Hygiene, Hospital Epidemiology and Environmental Health, University of Leipzig Medical, Germany; 2 Department of Visceral, Transplant, Thoracic, and Vascular Surgery at University of Leipzig Medical, Germany

**Keywords:** catheter related infection, peripheral venous catheter, compliance assessment, compliance self-assessment, peripheral venous catheter quality index

## Abstract

**Objective:**

The risk of peripheral venous catheter (PVC) infections in inpatients is often underestimated, even if it is lower than that for central venous catheters. Guidelines for the prevention of PVC-associated infections describe the evidence-based management of PVCs. The aims of this study were the development of standardized methods for compliance assessment regarding PVC management and the evaluation of self-reported knowledge and implementations among healthcare providers regarding PVC care.

**Method:**

We developed a checklist based on the recommendation of the Commission of Hospital Hygiene and Infection Prevention at the Robert Koch Institute (KRINKO) Berlin for the standardized evaluation of PVC management. The following parameters were collected and evaluated: condition of the puncture site, condition of the bandage, presence of an extension set, presence of a plug, and documentation. The checklist was applied in 14 normal wards in 2019. After feedback of the ward staff on the results, it was applied again in 2020 in the same wards. For retrospective data analysis, we used a newly developed PVC-quality index. After the second evaluation in 2020, we carried out an anonymous survey among the healthcare providers.

**Results:**

The evaluation of 627 indwelling PVCs showed a significant increase in compliance related to the presence of an extension set (p=0.049) and documentation (p<0.001) in the 2nd year. The quality index increased in 12 out of 14 wards. The participants of the survey were aware of the in-house standard “Prevention of vascular catheter-associated infections”, with a mean score of 4.98 on a Likert scale (1=not aware, 7=completely aware). The main barrier to implementation of the preventive measures was the time factor. Survey participants were more aware of PVC placement than PVC care.

**Conclusion:**

The PVC quality index is a valuable tool for the assessment of compliance regarding PVC management in daily practice. Feedback from the ward staff on the results of compliance assessment improves PVC management, but the outcome is very heterogeneous.

## Zusammenfassung

**Zielsetzung:**

Das Infektionsrisiko von peripheren Venenkathetern (PVK) bei stationären Patienten wird, auch wenn es niedriger ist als das von zentralen Venenkathetern, häufig unterschätzt. Leitlinien zur Prävention von PVK-assoziierten Infektionen beinhalten Evidenz-basierte Maßnahmen zur Anlage, Pflege und Nutzung von liegenden PVK. Ziel dieser Studie war, ein Verfahren zur standardisierten Bewertung der Compliance im Umgang mit liegenden PVK zu entwickeln und zu testen. Außerdem sollte eine Selbsteinschätzung des Personals zu Kenntnis und Umsetzung der Präventionsmaßnahmen erhoben werden.

**Methode:**

Basierend auf den KRINKO-Empfehlungen wurde eine Checkliste zur standardisierten Bewertung der Umsetzung von Präventionsmaßnahmen bei liegenden PVK entwickelt. Folgende Parameter wurden erhoben und bewertet: Zustand der Punktionsstelle, Zustand des Verbands, Vorhandensein eines Extensionssets, Verschluss, Nutzung und Dokumentation der Maßnahmen. Die Checkliste wurde erstmals im Jahr 2019 auf 14 Normalstationen angewendet und nach Feedback der Ergebnisse erneut im Jahr 2020 auf denselben Stationen eingesetzt. Mithilfe eines neu entwickelten PVK-Qualitätsindex erfolgte ein retrospektiver Vergleich. Nach der 2. Bewertung wurde eine anonyme Mitarbeiterbefragung mittels Fragebogen durchgeführt.

**Ergebnisse:**

Bei der Bewertung von 627 liegenden PVK zeigte sich eine signifikante Steigerung der Compliance bezogen auf das Vorhandensein eines Extensionssets (p=0,049) und die Dokumentation (p<0,001) im 2. Jahr. Der Qualitätsindex ist auf 12 der 14 beobachteten Stationen gestiegen. Die Bekanntheit einzelner Maßnahmen des hausinternen Standards „Prävention gefäßkatheterassoziierter Infektionen“ wurde mit dem Mittelwert 4,98 von 7 auf einer Likert-Skala angegeben; als einziges Hindernis in der Umsetzung konnte der Faktor Zeit identifiziert werden. Maßnahmen bei PVK-Anlage waren zu einem höheren Anteil bekannt als Maßnahmen der PVK-Pflege.

**Fazit:**

Der PVK-Qualitätsindex ist erfolgreich angewendet worden und ist geeignet, die PVK-bezogene Compliance standardisiert zu erheben. Feedback der Compliance im Umgang mit PVK hat diese verbessert, aber der Effekt ist sehr heterogen.

## Introduction

The use of venous catheters is a routine part of inpatient care. They are used for fluid therapy, intravenous administration of medication, and transfusions. There are central venous catheters (CVC) and peripheral venous catheters (PVC). PVCs are one of the most commonly used medical devices worldwide [1], [2]. According to Zingg et al. [3], up to 70% of all hospitalized patients have at least one PVC in situ. However, the use of vascular catheters carries the risk of catheter-associated infections. Up to now, the CVC has been seen as the more invasive device, which is why catheter-associated infections (especially sepsis) have been perceived as a risk primarily in connection with CVCs. However, more recent studies have shown that the use of PVC also represents a relevant risk due to the frequency of use [4]. These complications can be caused by inflammation at the entry site [5] or bacterially superinfected thrombophlebitis [6]. According to Maki et al., the infection rates in patients with a PVC is 0.5 per 1,000 PVC-days [7]. In a 7-year descriptive retrospective study. Ruiz-Giardin et al. [8] identified 285 patients with catheter-related bacteremia among 1,866 cases with bloodstream infections: 220 cases (77.19%) were caused by central venous catheter and 65 cases (22.81%) were associated with PVC. Appropriate PVC management in accordance with the guidelines can reduce the number of PVC-related complications. For instance, Bruno et al. [9] showed that the successful implementation of preventive measures reduced the incidence of PVC-related *S. aureus* bacteremia from 0.09 to 0.019 per 1,000 PVC-days. To prevent nosocomial vascular catheter-related infections, the Commission for Hospital Hygiene and Infection Prevention at the Robert Koch Institute (KRINKO) published recommendations in 2017 [10]. These recommendations include preventive measures during PVC insertion and ongoing PVC care [10]. The assessment of compliance regarding PVC management is also mentioned in KRINKO recommendations [10]. However, this important preventive measure is not standardized. According to § 23 (3) of the Infection Protection Act (IfSG) [11], the hospital’s health administration is responsible for the implementation of preventive measures and the assessment of adherence to the guidelines and recommendations. The aim of this study was to evaluate adherence to the guidelines and recommendations regarding PVC management [11] on 14 normal wards of a university hospital and to compare the results between the observed wards (part 1). In addition, we assessed the self-reported knowledge and self-assessed compliance of the staff on the same wards regarding PVC management (part 2).

## Methods

### Part 1a: Adherence to the guidelines regarding PVC management on 14 normal wards (retrospective analysis of PVC observations 2019–2020)

To evaluate adherence to the guidelines regarding PVC management, we developed a checklist based on the KRINKO recommendations [10] in 2018 at the Institute for Hygiene, Hospital Hygiene and Environmental Medicine of the University Hospital Leipzig. The checklist was first introduced in 2019, and includes seven quality indicators, which enable a standardized retrospective analysis of PVC data. We compared the checklist data of 14 selected adult wards from 2020 with the data from 2019. Between the observations in 2019 and 2020, we performed a feedback intervention: we presented the analyzed PVC data of each observed ward to the ward staff, and we made recommendations for improving the quality of PVC care. 

### Part 1b: Comparison of the observed wards

Based on the checklist parameters, we designed an instrument (PVC quality index [PVC-QI]) to enable the comparison of the observed wards at the different time points as well the comparison between the wards. Parameters of the checklist were defined as positive (compliant with the guidelines) or negative (non-compliant with the guidelines). The following parameters complied with the guidelines: presence of an extension line, presence of a Luer lock plug, absence of unnecessary PVC, absence of insertion-site complications, clean PVC dressing, stable PVC dressing; appropriate documentation in the medical records using one of the following systems: Copra^®^ from Copra System GmbH (Berlin, Germany), Care Complex Measures Score (PKMS) in SAP^®^ (ERP 6.0 from SAP Germany SE & Co. KG, Walldorf, Germany), Hinz^®^ Paper documentation by Hinz Fabrik GmbH (Berlin, Germany). The following parameters did not comply with the guidelines: absence of an extension line, absence of a Luer lock plug, unnecessary PVC (not accessed within the prior 24 hours), insertion-site complications (extravasation, erythema, swelling, heat, pain near insertion site), PVC dressing became soiled or damp, PVC dressing loosened, lack of documentation in the medical records. Based on these parameters, we developed a scoring system for the calculation of the quality index, where each parameter can receive 1 positive or 1 negative point, and PVC-QI is the ratio between all positive points and all negative points per year. An example of the PVC-QI calculation for the ward “N 1” is shown in Table 1 (Tab. 1): 1. In 2019, we evaluated 20 PVCs on this ward and all catheters had an extension line. Thus, the parameter “extension line” received 20 positive points. 2. In 2020, we did not find the appropriate documentation for three PVCs. Thus, the parameter “documentation” received three negative points. Based on all positive and all negative parameters, ward N1 had following PVC-QI:

- PVC-Quotient ward N1 (2019)=121/18=6.72
- PVC-Quotient ward N1 (2020)=74/11=6.73

Because the number of observed PVCs varied between the wards and within the same ward with time, we a corrected the PVC-QI accordingly. We included the number of PVCs observations in the PVC-QI calculation, so the increase in the number of observations results in an increase in PVC-QI. We multiplied the number of observations by 0.05 to achieve the optimal “reward” for the ward’s staff compliance during our audit and the ongoing PVC care, so that good results (high PVC-QI) could not be achieved simply by a large number of observations (maximal PVC-QI increase to 1/3 of the original value). Therefore, the mean number of observations should be 20 per ward to reach significantly higher PVC-QI. 

Formula for PVC-QI calculation:


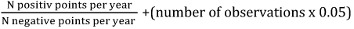


Based on correction ward N1 had following PVC-QI:

- PVC-QI ward N1 (2019)=121/18+(20x0.05)=7.72
- PVC-QI ward N1 (2020)=74/11+(17x0.05)=7.58

Higher PVC-QI corresponds with higher adherence to the guidelines and recommendations regarding PVC management.

### Part 2: Self-reported knowledge and self-assessed compliance of the ward staff regarding PVC management (survey)

After the audit, we performed an anonymous self-assessment survey of the ward staff to find the reasons for the lack of adherence to the guidelines not discovered during the PVC observations. We designed a two-page questionnaire (supplementary material) which included the following sections: person (age, gender, qualification), awareness of the in-house standard “Prevention of infection associated with venous catheter” and its influence on the daily practice, self-assessment regarding ongoing PVC care, including obstacles for the implementation of preventive measures in accordance with the guidelines. The awareness of the in-house hygienic standards and the self-assessment regarding the influence of the guidelines on the ongoing PVC care were assessed using a 7-point Likert scale ranging from 1=“absolutely inappropriate” to 7=“absolutely appropriate”. Awareness of the preventive measures during PVC insertion and ongoing PVC care was assessed with a yes/no question. Self-response regarding PVC management in accordance with the guidelines was assessed as a percentage.

The research protocol was approved by the locally appointed ethics committee at the Leipzig University, Faculty of Medicine N141/21-ek from 04/14/2021.

### Statistics

We used descriptive statistics, means and confidence intervals, to analyze the collected data. Missing values, e.g., due to incomplete checklists, were not included in the statistical analysis. We used Fisher's exact test to compare adherence to the guidelines and recommendations regarding PVC management on the observed wards in 2019 and 2020. This test was also used to analyze the differences between the self-assessed knowledge of preventive measures during PVC insertion and ongoing PVC care. Differences in PVC-QI between the observed wards were analyzed for statistical significance with the paired t-test. A p-value <0.05 was considered as statistically significant. For statistical analysis, we used GraphPad Prism version 9 (GraphPad Software, Inc., San Diego, California, USA). 

## Results

### Part 1a: Adherence to the guidelines regarding PVC management on 14 normal wards

Valid data were available for 332 PVC observations from 2019 and 295 observations from 2020 at the same 14 adult wards. The minimum cut-off for inclusion in the analysis was 10 observations. We found a significant increase in compliance regarding the presence of an extension line (p=0.049) and improvement in documentation management (p<0.001) in 2020 (after the feedback intervention) compared to 2019 (before the intervention). Table 2 (Tab. 2) shows adherence to the guidelines regarding PVC management on all observed wards.

### Part 1b: Comparison of the observed wards

Concordant with the previous finding (improvement in adherence to guidelines), we found a significant increase in PVC-QI on the observed wards: 12 out of 14 wards showed a significant positive trend. Table 1 (Tab. 1) presents an example of the PVC-QI calculation of the observed ward N1. Table 3 (Tab. 3) shows the differences in PVC-QI between the observed wards. The mean PVC-QI in 2019 was 4.7, whereas in 2020, it was 6.6 (p<0.003). Wards N1 and N7 showed a negative PVC-QI trend. Ward N7 showed the lowest PVC-QI: 1.85 in 2019 and 2.18 in 2020. Ward N10 also showed low PVC-QI, but the trend in 2020 was positive. Thus, PVC-QI enables comparison between the observed wards. Based on the PVC-QI data, more attention should be paid to ward N7 to improve PVC management in the next year.

### Part 2: Self-assessed compliance of the ward staff regarding PVC management (analysis of the feedback survey)

A total of 82 employees of the observed 14 wards were involved in the anonymous survey. 70.7% were female (N=58) and 29.3% male (N=24). The age ranged from 18 to 60 years, and the majority of participants (80.5%; N=66) was 18-40 years old. The largest subgroup (70.7%) were nurses, followed by doctors (24.4%) and a not specified subgroup (4.9%). The survey results regarding the preventive measures during PVC management are summarized in Table 4 (Tab. 4). Awareness of the in-house standard “Prevention of infection associated with venous catheter” was rated with a mean of 4.98. Self-assessed knowledge of preventive measures was rated with a mean 4.94. The respondents were less informed about the PVC audit and our feedback intervention, the mean values of which were 4.35 and 3.69, respectively. A daily check for unnecessary PVC was performed more often by nursing staff (5.48) than by doctors (4.32). The participants rated the correct implementation of the preventive measures as very important in order to avoid vascular catheter-associated infections (5.89) (Table 5 (Tab. 5)). All required materials were available to respondents (5.42). The respondents sometimes did not have enough time to implement the preventive measures effectively (4.08). The training on how to apply the preventive measures in accordance with the guidelines should also be improved (4.47). The respondents did not have enough time to learn the current guidelines and recommendations (4.51).

**Figure 1:**
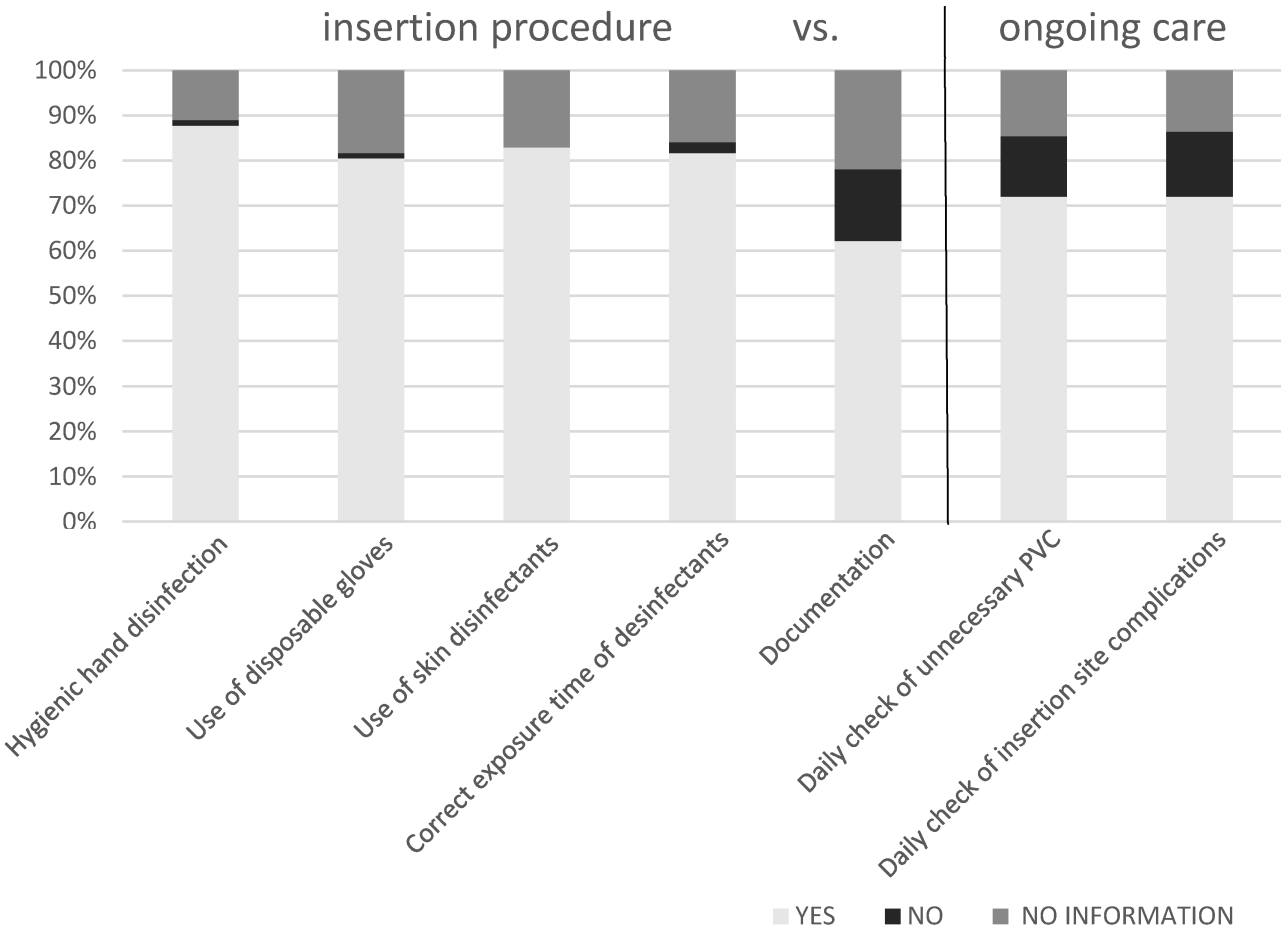
Awareness of the preventive measures during the PVC insertion procedure and the ongoing PVC care

The respondents were aware of the preventive measures during PVC insertion and ongoing PVC care. More than 80% of the participants knew the preventive measures during PVC insertion: hygienic hand disinfection according to the 5 indications of the WHO (87.8% of the participants), use of disposable gloves (80.5% of the participants), use of skin disinfectants (82.9% of participants) and the correct exposure time (81.7% of participants). Only 62.2% of the participants were aware of the documentation regarding PVC insertion. 72% of employees knew the preventive measures regarding ongoing PVC care, e.g., daily check for unnecessary PVC and insertion site complications,. The differences between the self-assessed knowledge of preventive measures during PVC insertion and ongoing PVC care were statistically significant (p=0.019). The results are shown in Figure 1 (Fig. 1).

## Discussion

According to § 23 (3) of IfSG [11], medical facilities should ensure that the required measures are performed for prevention of nosocomial infections in accordance with scientific evidence [11]. They are also responsible for the surveillance of nosocomial infections [11], such as infections associated with vascular catheters. This surveillance should be performed individually, because the assessment of all clinic departments is impossible due to restricted capacities [12]. High rates of nosocomial infections and high rates of procedures (catheter-days) can serve as selection criteria for surveillance. The assessment of adherence to the guidelines with PVC-QI on several wards requires effort, but this effort is significantly less than that required for primary surveillance. PVC-QI allows the identification of wards with low adherence to the guidelines and selection of these wards for additional training or targeted surveillance. The present study shows that the feedback intervention regarding adherence to the guidelines improved PVC management. In our study, 2 parameters were significantly improved: presence of an extension line (p=0.0485) and documentation (p<0.0001). Furthermore, 12 out of 14 wards showed a significant positive trend regarding PVC-QI. Wards N1 and N7 showed a negative PVC-QI trend. Ward N7 showed the lowest PVC-QI: 1.85 in 2019 and 2.18 in 2020. Thus, training of staff on these wards is necessary to eliminate ward-related obstacles to the implementation of preventive measures in accordance with the guidelines. The inclusion of these wards in surveillance is also legitimate. The compliance survey showed that the participants are aware of the preventive measures. The participants declared that the correct implementation of the preventive measures is important to avoid the vascular catheter-associated infections. On the other hand, they mentioned gaps in knowledge regarding preventive measures during ongoing PVC care, such as the daily check for unnecessary PVCs, insertion site complications and appropriate documentation.

### Limitations

First, the study was retrospective, and it included only 14 of 31 normal hospital wards (excluding pediatric and psychiatric wards). Therefore, the extrapolation of these data to other health facilities is possible only to a limited extent. Second, we did not assess the influence of adherence to the guidelines on the rates of PVC-associated local and bloodstream infections. Thus, the epidemiological relevance of the study is limited.

## Conclusions

Adherence to the guidelines is an important quality parameter. The assessment and monitoring of this parameter uncovers non-compliancy with the guidelines on PVC management, which is an important risk factor for PVC-associated infections. Thus, this parameter can serve as a tool for the prevention of bloodstream infections. The standardized method of compliance assessment regarding PVC management can improve data analysis and representation of the results to increase the adherence of healthcare personnel to preventive measures. The study showed that adherence to the guidelines regarding PVC management improved after the feedback intervention, but it is still not sufficient. PVC-QI allowed the identification of wards with low compliance regarding PVC management and thus selection of these wards for additional training and, if necessary, infection surveillance. More attention should be paid to the fact that the survey participants were better acquainted with the preventive measures during PVC insertion that those during ongoing PVC care. This knowledge should be taken into account when designing future training.

## Notes

### Competing interests

The authors declare that they have no competing interests.

### Acknowledgments

The authors would like to thank the Clinical Trial Center (ZKS Leipzig), Leipzig University, Faculty of Medicine for the statistical advice.

## Figures and Tables

**Table 1:**
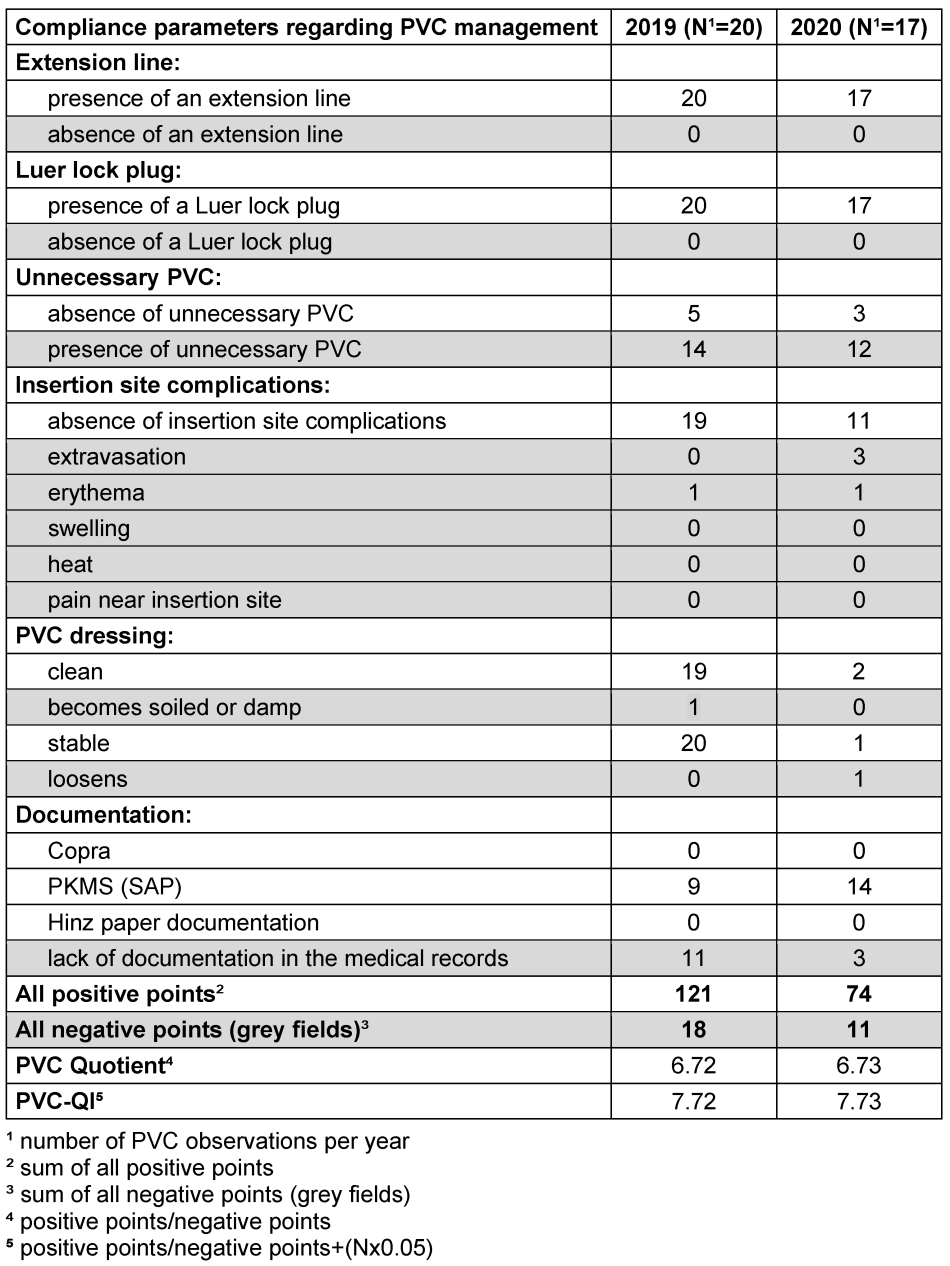
PVC-QI calculation of the observed ward N1

**Table 2:**
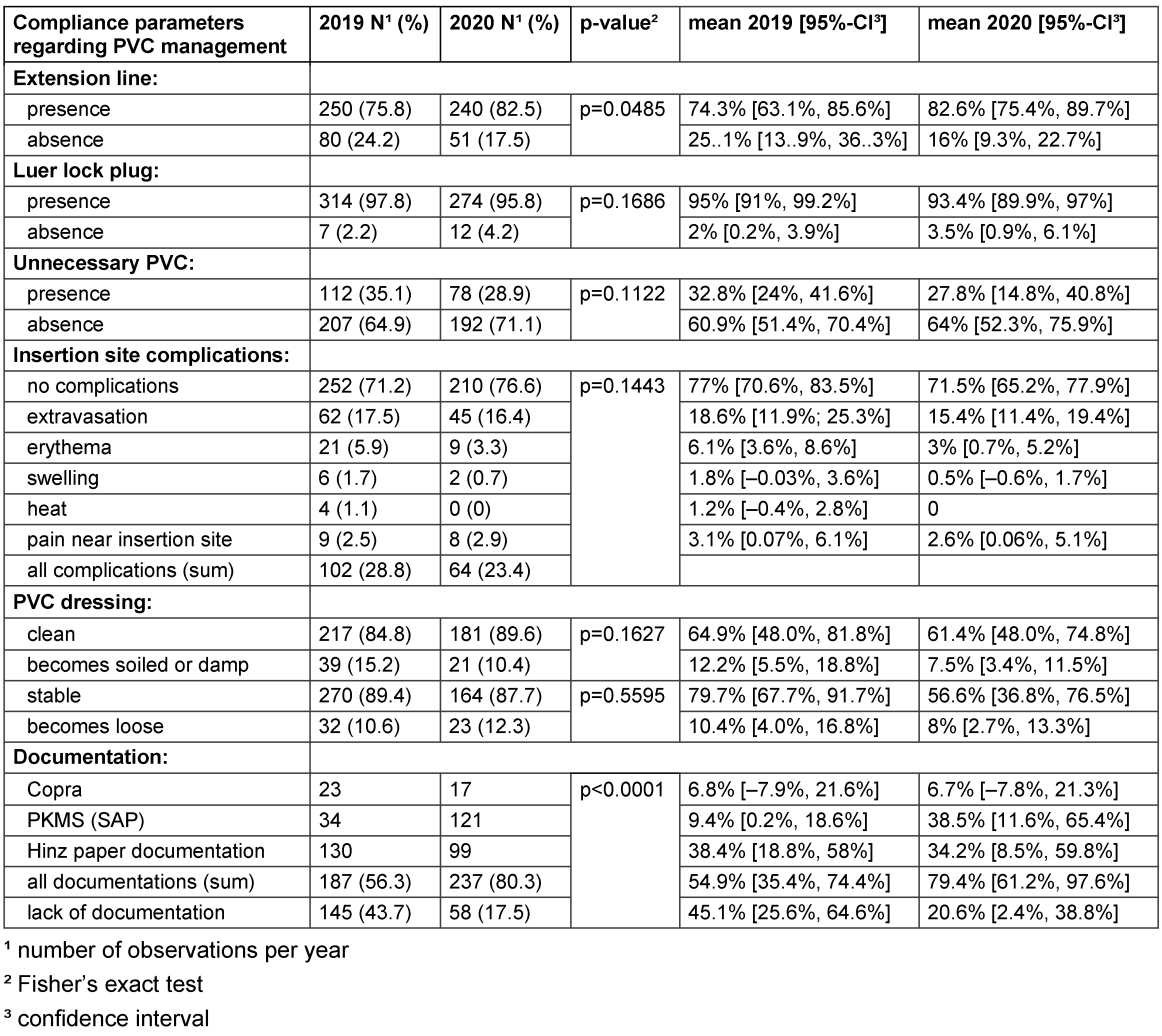
Adherence to the guidelines regarding PVC management on 14 normal wards

**Table 3:**
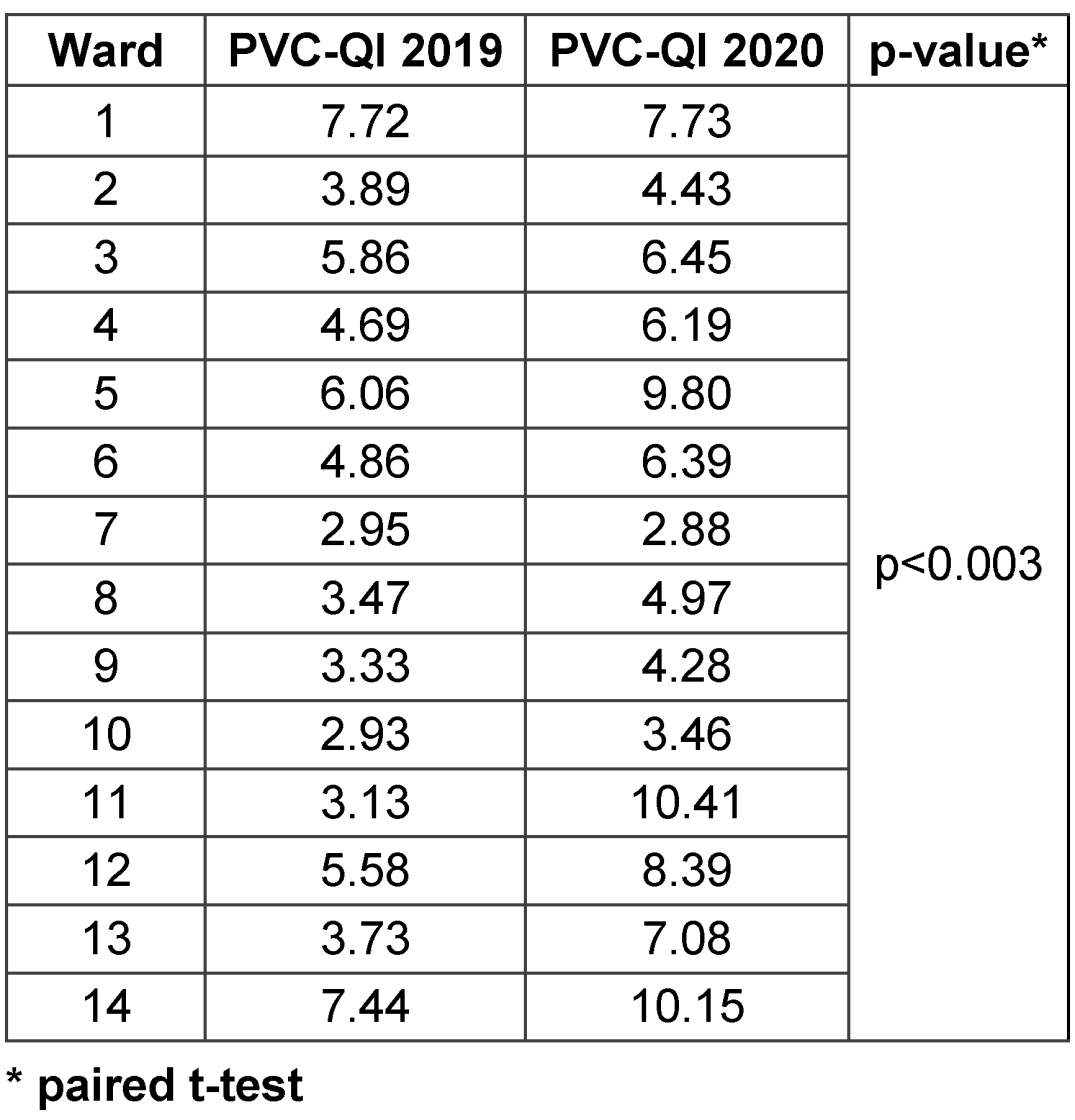
Differences in PVC-QI between the observed wards

**Table 4:**
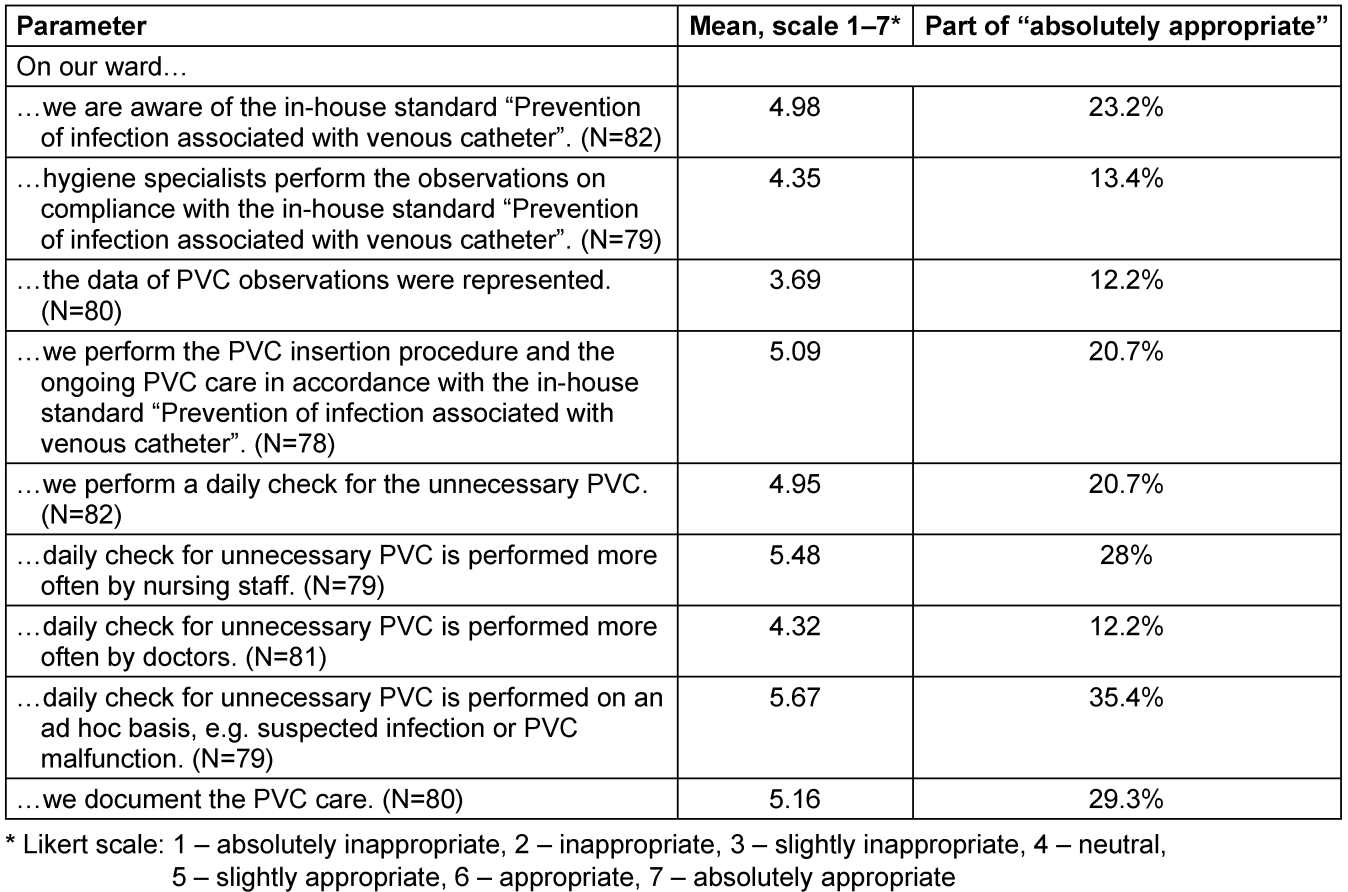
Self-assessment of preventive measures during the PVC management

**Table 5:**
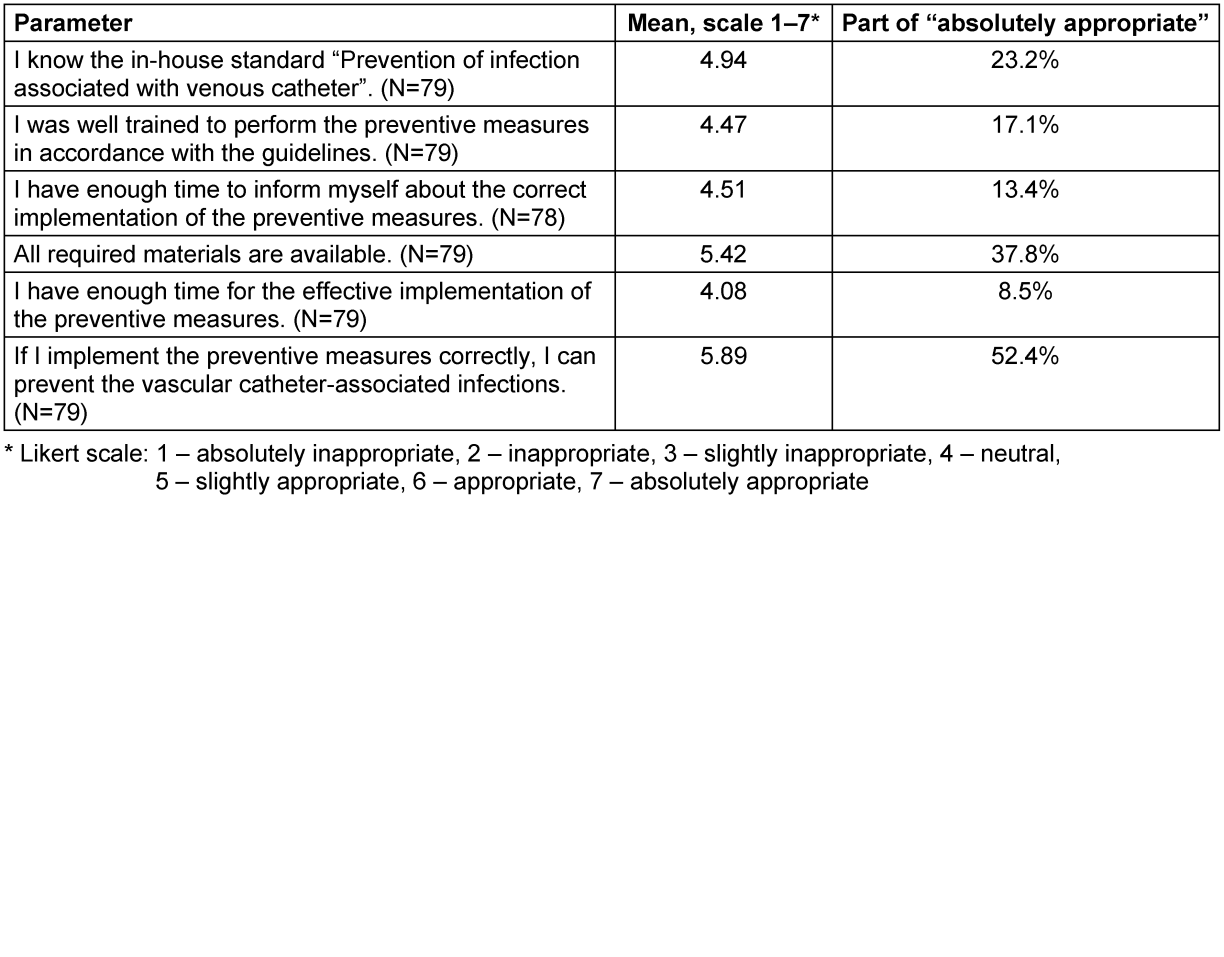
Self-assessed obstacles for the implementation of preventive measures in accordance with the guidelines
